# Metabolic engineering of *Bacillus subtilis* for growth on overflow metabolites

**DOI:** 10.1186/1475-2859-12-72

**Published:** 2013-07-25

**Authors:** Johannes Kabisch, Isabel Pratzka, Hanna Meyer, Dirk Albrecht, Michael Lalk, Armin Ehrenreich, Thomas Schweder

**Affiliations:** 1 Pharmaceutical Biotechnology, Institute of Pharmacy, Ernst-Moritz-Arndt-Universität, Felix-Hausdorff-Str. 3, D-17487 Greifswald, Germany; 2 Institute of Biochemistry, Ernst-Moritz-Arndt-Universität, Felix-Hausdorff-Str. 4, D-17487 Greifswald, Germany; 3 Institute for Microbiology, Ernst-Moritz-Arndt-Universität, Friedrich-Ludwig-Jahn-Str. 15, D-17487 Greifswald, Germany; 4 Department of Microbiology, Technische Universität München, Emil-Ramann-Str. 4, D-85354 Freising, Germany

**Keywords:** *Bacillus subtilis*, Metabolic engineering, Glyoxylate cycle, Expression system, Fed-batch, Acetate, Glycolic acid

## Abstract

**Background:**

The genome of the important industrial host *Bacillus subtilis* does not encode the glyoxylate shunt, which is necessary to utilize overflow metabolites, like acetate or acetoin, as carbon source. In this study, the operon encoding the isocitrate lyase (*aceB*) and malate synthase (*aceA*) from *Bacillus licheniformis* was transferred into the chromosome of *B. subtilis.* The resulting strain was examined in respect to growth characteristics and qualities as an expression host.

**Results:**

Our results show that the modified *B. subtilis* strain is able to grow on the C2 compound acetate. A combined transcript, protein and metabolite analysis indicated a functional expression of the native glyoxylate shunt of *B. lichenifomis* in *B. subtilis*. This metabolically engineered strain revealed better growth behavior and an improved activity of an acetoin-controlled expression system.

**Conclusions:**

The glyoxylate shunt of *B. licheniformis* can be functionally transferred to *B. subtilis*. This novel strain offers improved properties for industrial applications, such as growth on additional carbon sources and a greater robustness towards excess glucose feeding.

## Background

The anaplerotic glyoxylate cycle is a variation of the tricarboxylic acid cycle (TCA cycle), which allows acetyl-CoA to be used to replenish carbon for anabolic processes [1]. The isocitrate lyase of this shunt prevents a carbon loss by cleaving isocitrate into glyoxylate and succinate, thus avoiding the decarboxylation of isocitrate and 2-oxoglutarate. Glyoxylate and acetyl-CoA are joined to malate by the malate synthase, the second enzyme of the glyoxylate shunt, which allows for the regeneration of oxaloacetate that is henceforth available for anabolic activities via the TCA cycle or gluconeogenesis. From a bioprocessing viewpoint, this ability is especially interesting for bacteria that previously produced overflow metabolites. In terms of *Bacillus subtilis* such conditions include aerobic growth with excess glucose or anaerobic nitrate respiration [2,3] and lead to the formation of acetate as well as acetoin and 2,3-butanediol. Industrial fermentation processes are usually so designed that overflow metabolism is avoided or at least limited. The overflow metabolism leads to metabolic shifts towards less efficient pathways and causes energy spilling reactions [3]. Nevertheless the mixing of incoming, highly concentrated feed solutions is insufficient in industrial-scale bioreactors and always gives rise to zones of excess carbon source [4]. For *Escherichia coli* it has been shown that the formation of overflow metabolites, especially acetate, hampers large scale fed-batch processes. Accumulated acetate has been observed to have adverse effects on target product formation and cell growth [5].

After depletion of the preferred carbon source (e.g. glucose), the excreted overflow metabolites are converted into metabolically utilizable acetyl-CoA [6]. Since *B. subtilis* lacks the capability to conduct the glyoxylate cycle, it can only make use of the energy provided by the overflow metabolites in the form of reduction equivalents, which are generated during the TCA cycle. In this study the transfer of the glyoxylate shunt - operon from its close relative *Bacillus licheniformis*[7] was investigated in *B. subtilis.* It is shown that the functional expression of this operon enables *B. subtilis* to utilize the overflow metabolite acetate as a carbon source and resulted in a more robust growth behavior with excess glucose and an increased production of a recombinant reporter enzyme.

## Results

**Figure 1:**
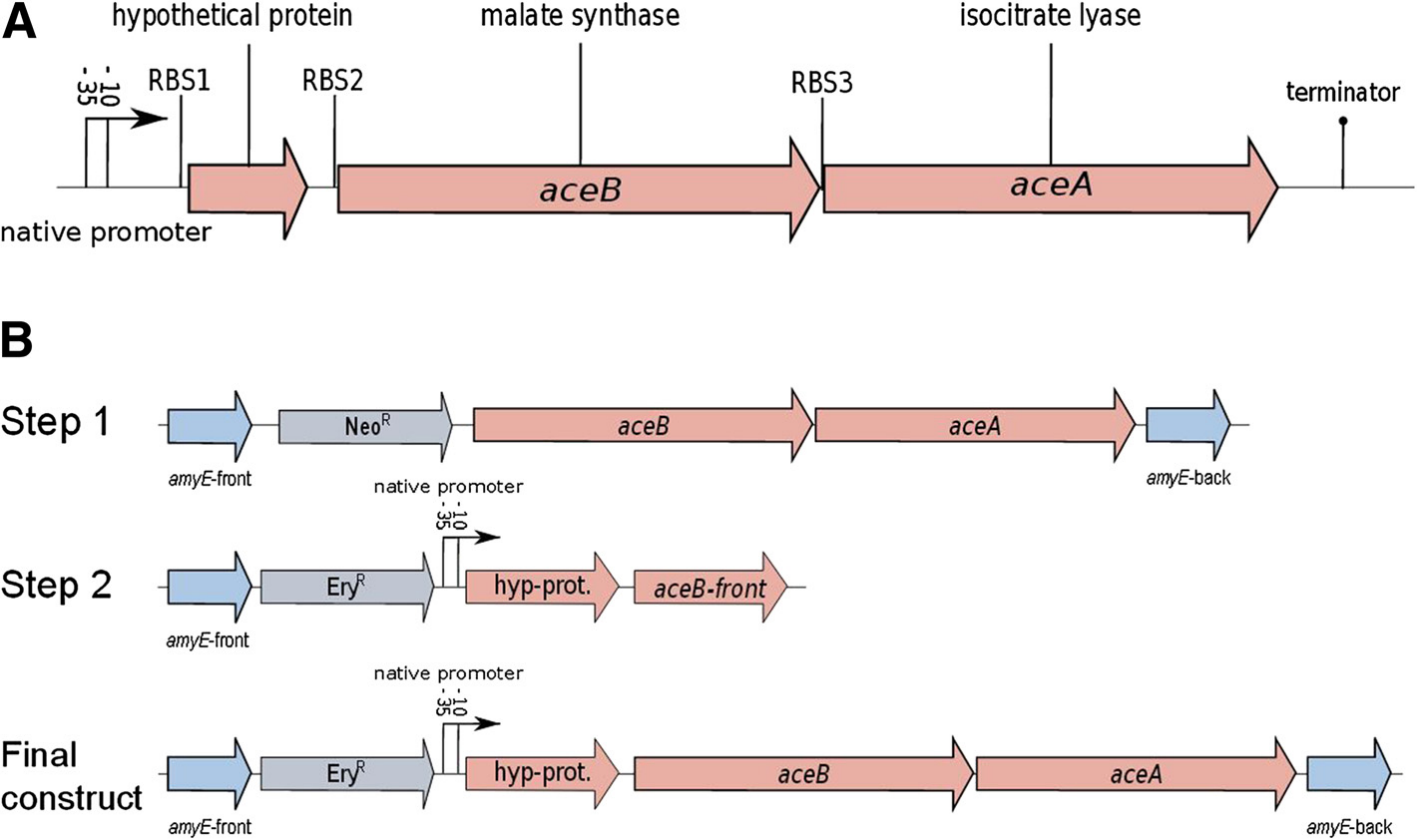
**Structure of the *aceBA***-**operon of *B. licheniformis *(A) and schematic representation of its transfer into the *B. subtilis *genome (B). (A)** The *aceBA*-operon of *B. licheniformis* DSM13 encodes the malate synthase (*aceB*), the isocitrate lyase (*aceA*) and a hypothetical protein which is essential for the functional transfer of this operon in *B. subtilis.* (RBS: ribosome binding site). **(B)** In a first step, the *aceB* and *aceA* genes without a promoter but a neomycin resistance cassette (Neo^R^) were integrated into the chromosomal *amyE*-locus of *B. subtilis* by a double cross-over event. In a second step the resulting strain was transformed with a DNA-fragment containing the gene of the hypothetical protein and the native promoter of the *aceBA*-operon by selecting on the erythromycin resistance cassette (Ery^R^).

Primer extension experiments with *B. licheniformis* showed that the transcript of the glyoxylate operon (*aceBA*) starts 231 bp upstream of the *aceB* gene with a potential promoter sequence at the position of 4,040,629 to 4,040,601 in the genome (personal communication A. Ehrenreich). Analysis of the 3’-end with the Vienna RNA package [8] revealed a potential terminator structure (4,037,415 to 4,037,434; –∆G = 15.2) and typical Shine-Dalgarno sequences (SDS) in front of a hypothetical protein sequence (RBS1: 4,040,515 to 4,038,510), *aceB* (RBS2: 4,040,338 to 4,040,332) and *aceA* (RBS3: 4,038,731 to 4,038,726) (Figure 1A). These SDS meet the requirements for an efficient ribosomal binding side of *B. subtilis*[9].

First attempts to construct plasmids containing the complete glyoxylate operon of *B. licheniformis* failed with *E. coli* as subcloning host. Transformation of the ligation reaction using *E. coli* as a shuttle host resulted in plasmids containing different fragments of the genes, but never the complete operon, making it likely that ORFs of the glyoxylate operon are expressed in *E. coli* and interfere with its metabolism. Therefore, we constructed the plasmids in such a manner that one plasmid contained only the *aceB* and the *aceA* genes without a promoter and another plasmid contained the native *aceBA*-operon specific promoter and the above mentioned hypothetical protein (Figure 1B). Both plasmids could be propagated in *E. coli* and the transformation of *B. subtilis* in a two-step-procedure worked well, yielding *B. subtilis* ACE which contains the native glyoxylate operon of *B. licheniformis* in its chromosomal *amyE* locus.

**Figure 2:**
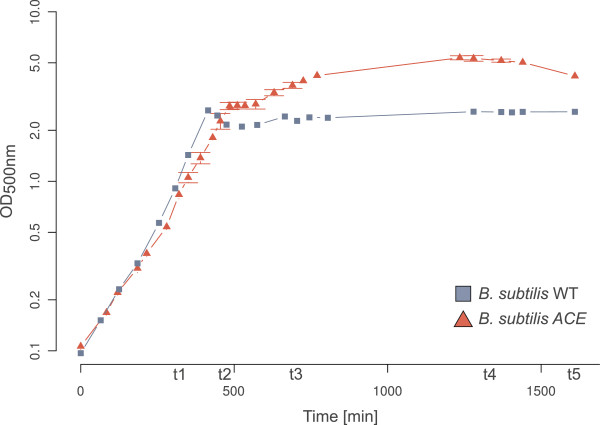
**Growth comparison of the *B. subtilis *WT and the glyoxylate shunt - expressing ACE strain. **The cultivations were performed in three biological replicates in mineral salt medium (MSM) containing 0.08% glycerol, 0.2% acetoin and 0.5% acetate. t1-t5 indicate sampling points.

In mineral salt medium containing a mixture of glycerol, acetate and acetoin as carbon sources, the glyoxylate-positive strain *B. subtilis* ACE reached a significantly higher final maximal optical density than the wild type strain (Figure 2). Glycerol is in this medium the preferred carbon and energy source for *B. subtilis*. In contrast to the wild type (WT) *B. subtilis* ACE showed a diauxic growth pattern with a slightly reduced specific growth rate during the mid-exponential growth phase, indicating a utilization of acetate but also acetoin for its growth.

**Figure 3:**
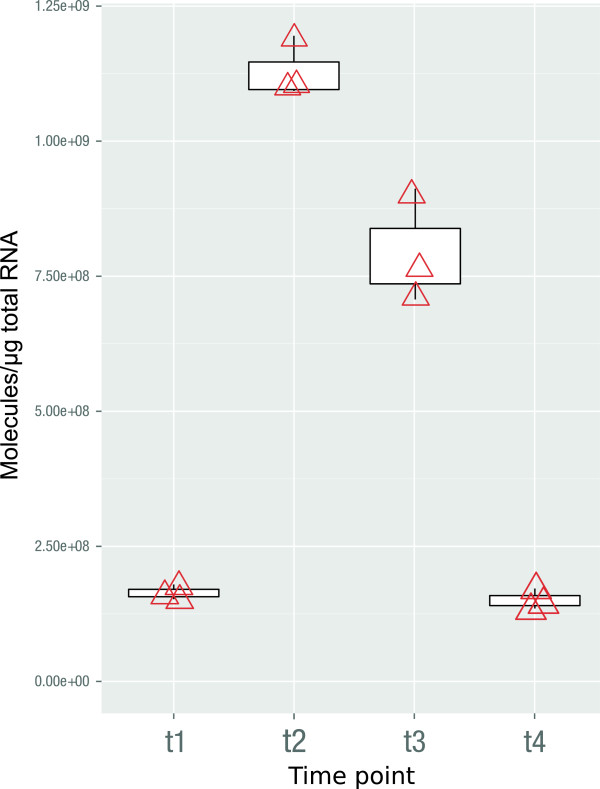
***aceB-*mRNA quantification by real-time-RT-PCR. **The length of the detected *aceB*-transcript was 300 nucleotides. ACE = *B. subtilis* encoding the *aceBA*-operon. t1-t4 sampling points throughout the growth curve as shown in Figure 2. N = 3, therefore the box plot does not show quartils.

A quantitative *aceB*-mRNA-analysis indicated a basal expression of the *aceBA*-operon in *B. subtilis* ACE during the exponential growth on glycerol (Figure 3). A significant induction (~7-fold) occurred during the diauxic transition phase. The *B. subtilis* control strain (WT) did not show a detectable *aceB*-mRNA signal in any of the experiments (data not shown). The expression of the isocitrate lyase (AceA) in the *aceBA* positive strain was detectable in the cytoplasmic soluble proteome during the transient and stationary phase by mass spectrometry analyses. No AceA-specific peptides could be identified in the negative control (WT). However, no peptides could be detected for the malate synthase (AceB) and the hypothetical protein in any of the samples. The difficulty of the identification of AceB-specific peptides was not unexpected, since the protein has so far eluded identification in several gel-based studies of *B. licheniformis* (personal communication B. Voigt, [10]). With a molecular weight of about 5 kDa, the absence of peptides for the hypothetical protein can be explained by the difficulties of analyzing such small proteins with the gel-based MALDI-TOF mass spectrometry.

**Figure 4:**
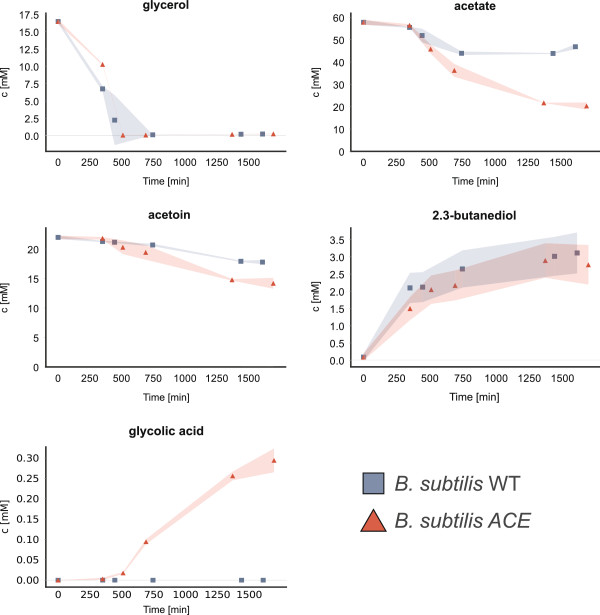
**Quantification of selected extracellular metabolites. **^1^H-NMR-quantified metabolites of the added carbon sources and selected byproducts of the *B. subtilis* wild type (WT) and the *B. subtilis* ACE strain with the glyoxylate cycle operon (ACE). The corresponding sampling points are indicated in Figure 2.

The analysis of extracellular metabolites revealed the consumption of glycerol as the preferred carbon source and a nearly constant level of acetoin and acetate during this first growth phase (Figure 4). Thus, in contrast to growth on glucose [6,11], *B. subtilis* does not produce overflow metabolites like acetate or acetoin in detectable amounts while using glycerol as main carbon source. Both strains showed a weak utilization of acetate and acetoin already during the late exponential growth phase on glycerol. However, the glyoxylate cyle - positive ACE strain revealed a significant higher utilization of acetate but also of acetoin compared to the WT. After exhaustion of glycerol the ACE strain started to accumulate glycolic acid. In contrast, no such accumulation could be detected in the *B. subtilis* WT strain. It is interesting to note that the final glycolic acid concentration in the *B. subtilis* ACE mutant strain was almost three times as high as that of the *aceBA*-donor strain *B. licheniformis* DSM13 (data not shown). Glyoxylate could not be detected in the supernatant of any strain. 2.3-butanediol accumulated in both *B. subtilis* strains and was not consumed (Figure 4).

**Figure 5:**
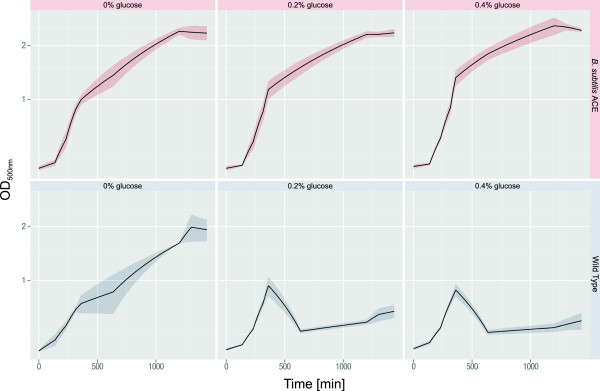
**Simulated of fed-batch and high glucose conditions with EnBase Flo. **Wild type = *B. subtilis ΔamyE; B. subtilis* ACE = glyoxylate cycle - positive mutant with the *aceBA*-operon integrated into the *amyE*-locus. The final optical densities (OD) were measured with a 1 cm path length cuvette with the following results: WT 0% glucose = 8.77; WT 0.2% glucose = 2.05; WT 0.4% glucose = 1.85; ACE 0% glucose = 15.18; ACE 0.2% glucose = 16.16; ACE 0.4% glucose = 16.84*.*

Simulated fed-batch cultivation experiments using ‘EnBase Flo’ revealed a clear distinction of growth patterns between the WT and the glyxolate cycle - positive mutant (Figure 5). *B. subtilis* ACE reached higher cell densities and was not negatively affected by the addition of excess glucose. In contrast, the WT only exhibited a relative stable growth behavior without additional glucose. The excess of glucose led to a lysis of the WT cells after 360 min of incubation. All cultures, except the WT cultures with 0.2 or 0.4% glucose, featured a pronounced biofilm formation in the late growth phase (at about 1200 min). Additionally, *B. subtilis* ACE produced a red pigment, which was not detectable in the WT cultures. The formation of biofilms and pigments hampered the exact determination of the optical density in these experiments. However, the determined trend in the growth behavior was reproducible for all investigated biological replicates. The absorption maximum of the observed red pigment in these cultures was at 410 nm, while only a minor absorption could be observed at 500 nm [12]. Care was as well taken not to pipette the biofilm but only the cells underneath. It should be noted that due to different path lengths and measurement methods of the plate reader in comparison to a standard photometer, OD values shown in Figure 5 are relative values. The final ODs of the cultures measured with a standard 1 cm path length photometer are given in the legend of Figure 5.

**Figure 6:**
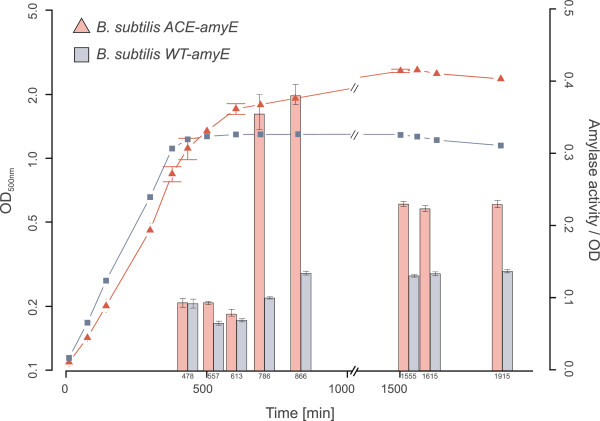
**Influence of the glyoxylate shunt on the *acoA*-controlled amylase overproduction of *B. subtilis *ACE compared to the WT. **In both strains the native alpha-amylase gene was disrupted and a functional copy under control of the *acoA*-promoter was integrated into the *sacA*-locus. Exact sampling points for activity measurements are indicated in small type. Medium: MSM + 0.5% acetate, 0.08% glycerol, 0.5% acetoin.

In order to further characterize the glyoxylate shunt - expressing *B. subtilis* strain, studies using an acetoin-inducible promoter (*acoA*) and a secreted alpha-amylase [13] as a reporter enzyme were carried out. In order to increase the transcription activity of the *acoA*-promoter the MSM growth medium was supplemented with 0.5% acetoin. Similar to the growth experiments shown in Figure 2 the ACE-strain revealed a higher final optical density in comparison to the WT (Figure 6). It is interesting to note that the *B. subtilis* ACE strain enables an increased *acoA*-directed amylase overproduction under these conditions. These experiments also indicate that the *acoA*-promoter is repressed during growth on glycerol [14] and is activated during the transient phase in the WT culture and during growth on acetate in the *B. subtilis* ACE strain after glycerol has been consumed.

## Discussion

Relatively little is known about the regulation of the glyoxylate cycle in Gram-positive bacteria. Results of this study indicate a control of the *aceBA*-operon of *B. licheniformis* in *B. subtilis* at the transcriptional level*.* A transcriptional regulation of the glyoxylate shunt has also been shown for the Gram-positive bacterium *Corynebacterium glutamicum*, where the expression of the malate synthase and isocitrate lyase is positively controlled by the LuxR-type transcriptional regulator RamA in the presence of acetate [15]. Voigt et al. detected a strong induction of the AceB expression in *B. licheniformis* at the transcriptional and translational level as soon as the preferred carbon and energy source glucose is exhausted [16]. Schroeter et al. [10] could determine an induction of the glyoxylate shunt during peroxide stress experiments, which was attributed to an irreversible oxidation of the isocitrate dehydrogenase (ICDH). However, it is also shown in our study that the region encoding the hypothetical protein (BL02643) in front of *aceBA* is essential for a functional transfer of the glyoxylate shunt to *B. subtilis*. Strain constructs missing this putative regulatory region of the operon fail to grow on acetate (data not shown). It can be concluded that this small protein plays an essential role in the regulation of the glyoxylate cycle activity.

The incorporation of the glyoxylate shunt - encoding operon led to growth of *B. subtilis* ACE on the overflow metabolite acetate and to a lesser degree on acetoin. A diauxic growth with a preferred utilization of glycerol over acetate can be derived from the metabolite analyses (Figure 4). However, the current version of the glyoxylate cycle - positive strain *B. subtilis* ACE failed to reach the same maximal optical density as *B. licheniformis* (data not shown). A possible reason for its earlier growth arrest might be the accumulation of glycolic acid (see Figure 4). This small alpha-hydroxy acid has been described as a potent inhibitor of the isocitrate lyase of *Pseudomonas indigofera*[17] and could disable *B. subtilis* ACE to continue utilizing acetate as a replenishing carbon source through its glyoxylate cycle. Glycolic acid is also formed in *B. licheniformis*, but to a much smaller extent (data not shown). *B. licheniformis* DSM13 possesses a putative glyoxylate reductase (ORF BL02138) which exhibits a 70% identity to a similar enzyme (*gyaR*) of *Bacillus pumilus* ATCC 7061. This putative glyoxylate reductase (ORF BL02138) could catalyze the reduction of glyoxylate to glycolic acid and could thus lead to a reduced accumulation of glycolic acid in *B. licheniformis*.

The accumulation of 2.3-butanediol in *B. subtilis* during the exponential and stationary phase is likely due to the activity of the acetoin reductase/2.3-butanediol dehydrogenase (*bdhA*) [18]. The remaining acetoin could block the reverse reaction that would transform the 2.3-butanediol back to acetoin. It has been reported that the presence of acetic acid leads to an increased production of 2.3-butanediol by *B. subtilis*[19]. In contrast, *B. licheniformis* is able to utilize acetoin but also 2.3-butanediol entirely (data not shown), which might also have contributed to an increase of biomass. This is most probably also supported by the butanediol/acetoin reductase encoded by the *budC* (BL01177) gene of *B. licheniformis*, which catalyzes the irreversible reduction of 2.3-butanediol to acetoin [20]. Interestingly, the subspecies *B. subtilis subsp. spizizenii,* which is of a different lineage than the used *B. subtilis* strain 6051HGW [21], encodes the same potential for the 2.3-butanediol utilization as *B. licheniformis*, highlighting both as potential donors of the butanediol/acetoin reductase for a further optimization of *B. subtilis* ACE.

The simulated EnBase fed-batch cultivation indicated an advantage of the glyoxylate shunt - active strain *B. subtilis* ACE in comparison to the WT under high glucose concentration conditions. Induction of a significant overflow metabolism by addition of excess glucose presumably resulted in the growth arrest of the WT, while the activity of the glyoxylate shunt enabled *B. subtilis* ACE to exploit too high levels of these metabolites and to reach thus elevated cell densities.

This study indicates that the previously described *acoA*-expression system [13] displays an improved performance in the glyoxylate cycle - positive strain *B. subtilis* ACE. The promoter of the *acoABCL*-operon is repressed by PTS–sugars [22], but as demonstrated in this study also by the non-PTS carbon source glycerol. The cheap C-source glycerol in combination with acetate therefore offers an interesting alternative for the control of the *acoA*-based *B. subtilis* expression system. Another possible application of *B. subtilis* ACE may be an auto-inducing system that depends on phosphate limitation. As described by Dauner et al*.*[3], *B. subtilis* produces large quantities of acetate during chemostat experiments with high glucose and low phosphate levels. The ability of *B. subtilis* ACE to produce higher recombinant target protein levels, while growing on acetate, would therefore be a suitable alternative possibility for the application of the *acoA*-expression system.

## Conclusion

The *aceBA* operon of *B. licheniformis* is a self-contained metabolic entity that can be functionally transferred to *B. subtilis*. The obtained strain is able to grow on acetate as carbon source which results in an increased biomass formation and an improved production of a recombinant model protein. In addition, the glyoxylate cycle-positive *B. subtilis* mutant cells feature greater robustness towards excess glucose cultivations.

## Methods

### Construction of plasmids, strains, and genome analysis

Chromosomal DNA of *Bacillus licheniformis* MW3, which is a mutant of *B. licheniformis* DSM13 [23], was used as template for the amplification of the *aceBA*-operon. Sequence information was derived from the published genome of *B. licheniformis* DSM13 [GenBank:NC_006322]. PCRs were performed with the Phusion polymerase. The utilized oligonucleotides are summarized in Table 1, while the constructed plasmids and used strains are listed in Table 2.

**Table 1:** Oligonucleotide primers used in this study

**Name**	**Sequence 5’ → 3’**	**Usage**
aceBAfor	GATCGTCTAGAGTCTCCCTACCCCTTTACAT	Forward primer for *aceBA* operon
aceBArev	GATCGGATCCAAGACAACCGGCCGGCCAAA	Reverse primer for *aceBA* operon
Prom1-for	GAAAATGGCTAAAATTGGTTATGCACGACTCTACGAAT	Forward primer for integration of native promoter
Prom1-rev	CGCAACGCGGGGAGGCAGACAAGGTATAGGGCGGGG ACCAAAATCGAACAGGCTTGC	Reverse primer for integration of native promoter
SLIC-Ery-for	TTATGCACGACTCTAGCCTGCAGGTCGACTGATAAGACGGTTCGTGTTCG	Forward primer for *Ery*^*R*^ cassette
SLIC-Ery-rev	TAATTAGACCGGTACCCGGGGATCCTCTAGCTCTTTAGCTCCTTGGAAGC	Forward primer for *Ery*^*R*^ cassette
aceB-for	CTGCCGCCGATCAGCACATGC	Forward primer for mRNA-standard of *aceB*
aceB-rev	CCCGCAAATTAACCTGGC	Reverse primer for mRNA-standard of *aceB*
aceB-rev-T7	TAATACGACTCACTATAGGGCCCGCAAATTAACCTGGC	Reverse primer for mRNA-standard of *aceB* with T7-promoter

**Table 2:** Strains and plasmids used in this study

**Strain**	**Genotype**	**Reference**
*B. subtilis* 6051HGW	Wild type	[24]
*B. licheniformis MW3*	*ΔhsdR1, ΔhsdR2*	[23]
*B. subtilis* ACE	Δ*amyE::aceBA*	This study
*B. subtilis* ACEProm1	Δ*amyE::aceBA*	This study
*B. subtilis* ΔamyE	Δ*amyE::Ery*^*R*^	This study
*B. subtilis* acoAamyE	Δ*amyE::Ery*^*R*^ Δ*sacA::acoAamyESSS*	This study
*B. subtilis* ACEamyE	Δ*amyE::aceBAProm1SSE* Δ*sacA::acoAamyESSS*	This study
*E. coli* DH10B	F-*mcrA,*(*mrr-hsdRMS-mcrBC*)’80*lacZ,*M15, *lacX74, recA1 araD139, (ara leu)* 7697	[25]
**Plasmid**	**Function**	**Reference**
pBGAB	Integration of genes into the *amyE* locus with Neo^R^	[26]
pACEBA	*aceBA* chromosomal integration into the *amyE* locus without promoter	This study
pMTL500	Source of *ermB* gene for erythromycin resistance	[27]
pAMYSSE	Integration of genes into the *amyE* locus with Ery^R^	This study
pProm1	Integrating of the native *aceBA* promoter and the gene encoding the hypothetical protein	This study
pJK168	Integration of genes into the *sacA*-locus	J. Kumpfmüller (unpublished results)
pMJS2	Source of the P_*acoA*_-*amyE* construct	[13]
pSacAmyE	Integration of the *amyE* gene under P_*acoA*_ control into the *sacA*-locus	This study

*Ery*^*R*^: Erythromycin resistance cassette, *Neo*^*R*^: Neomycin resistance cassette, *amyE*: Alpha-amylase gene, *P*_*acoA*_: Promoter of the *acoABCL*-operon, *sacA*: Phospho-sucrase gene.

The plasmid pACEBA was constructed by amplifying the *aceBA*-operon without its predicted promoter region and the hypothetical protein. The PCR product and the pBGAB [26] plasmid were cut with XbaI and BamHI and ligated using T4-ligase. To integrate the native promoter and the hypothetical protein in front of the *aceBA*-operon, the pBGAG plasmid was cut with SmaI and NotI and an erythromycin cassette from pMTL500 [27] was inserted yielding the plasmid pAMYSSE. Hereafter, this plasmid was cut with NarI and EcoRI and the PCR product resulting from the Prom1-primer pair was integrated via a modified sequence and ligation-independent cloning method [24]. Electroporation of the plasmids into *E. coli* was performed as previously described [28]. All utilized enzymes were purchased from New England Biolabs (Frankfurt a. M., Germany), oligonucleotides were synthesized by Life Technologies (Darmstadt, Germany).

To obtain an *aceBA*-encoding strain, *B. subtilis* 60-51HGW was transformed via natural competence [29] using the linearized plasmid pACEBA. The clones were selected on neomycin and the positive mutants were designated as *B. subtilis* ACE-P. To functionalize the operon, pProm1 was transformed in the same manner into *B. subtilis* ACE-P with erythromycin selection resulting in *B. subtilis* ACE. To examine the expression of a reporter enzyme, *B. subtilis* ACE and *B. subtilis* 6051HGW were transformed with pSacAmyE yielding a wild type (*B. subtilis* WTamyE) and a glyoxylate cycle - positive mutant (*B. subtilis* ACEamyE) that express and secrete an alpha-amylase under control of the *acoA*-promoter [13]. pSacAmyE was constructed by cutting pJK168 and pMJS2 [13] with XbaI and ligating the *acoA-amyE* fragment from pMJS2 into the *sacA* landing pads of pJK168. To ensure comparable alpha-amylase measurements, the native alpha-amylase of *B. subtilis* was disrupted by the integration of the *aceBA-*operon (*B. subtilis* ACEamyE) and an erythromycin cassette of plasmid pAMYSSE (*B. subtilis* WTamyE) into the *amyE*-locus. All plasmid constructs and chromosomal integrations were verified by sequencing.

The publicly available genome sequence of *B. licheniformis* was annotated in 2004 [7]. Since then, a large amount of novel sequences and annotations have been published, which implicates the necessity to update previous functional assignments for organisms of interest. Accordingly, we reannotated the genome sequence of *B. licheniformis* DSM13 in the same manner as described for *B. subtilis* 6051HGW [24]. In short, the GenBank file of *B. licheniformis* DSM13 [GenBank:AE017333] was imported into the GenDB 2.2 [30] pipeline and the resulting database was used to analyze the ORFs with JCoast [31].

### Cultivation conditions

Cultivations were carried out at 37°C and 250 rpm shaking in Erlenmeyer flasks with a maximum culture volume of one fifth of the flasks volume. Pre-cultures were inoculated from a cryo-stock and grown in LB medium for 5 h and then transferred into the appropriate medium for overnight cultures. Main cultures were inoculated from exponentially growing overnight cultures. The composition of the mineral salt medium (MSM) and its trace element solution has been described previously [24]. The MSM was supplemented with 0.08% glycerol, 0.5% acetate, 0.2% acetoin, and 2 mM MgSO_4_ if not indicated otherwise.

In order to test fed-batch like conditions, we cultivated the glyoxylate cycle - positive mutant and the WT strain with 3 ml EnBase Flo Liquid (# EBLM500, BioSilta Oy, Oulu, Finland) medium in 24-deep-well plates. The EnBase system is based on the slow release of glucose from a polymer through the action of an enzyme, thus acting like a substrate feeding pump [32]. The native amylase of *B. subtilis (amyE)* has been reported to interfere with this method (personal communication P. Neubauer). Therefore, *B. subtilis* amylase knock-out strains (Table 2) were used in all EnBase cultivations. Beside the control culture without external glucose addition, cultures supplemented with 0.2% and 0.4% glucose were investigated to simulate high glucose conditions and to stimulate acetate formation. 1.5 U of the EnBase-specific hydrolase (BioSilta Oy, Oulu, Finland) was used in all experiments. Plates were shaken at 300 rpm with an amplitude of 25 mm. Sample volume was 10 μl, with which the OD at 500 nm was determined in a 1:10 dilution in a Tecan infinite M200 plate reader (Männerdorf, CH).

### RNA analysis

RNA samples were taken and prepared as previously described [33]. An additional treatment with RNAse-free DNAse (#79254, Qiagen, Hilden, Germany) was performed followed by a purification with the RNA Clean-Up and Concentration Kit (#23600) from Norgen Biotek (Thorold, Canada).

The *aceB*-mRNA-standard was created with the primers aceB-for and aceB-rev-T7 (Table 1) using the DIG RNA Labeling Kit (SP6/T7) from Roche (Mannheim, Germany) and substituting the DIG-labeled nucleotides with unlabeled NTPs (Fermentas UAB, Vilnius, Lithuania).

The real-time RT-PCR analysis was performed as described by Hoi et al. [34]. The RNA samples were prepared as recommended by the supplier of the LightCycler® RNA Master SYBR Green I (#03064760001, Roche). The RT-PCR reaction was prepared with the primers aceB-for and aceB-rev (Table 1) and analyzed applying the Roche LightCycler as suggested by the manufacturer with an annealing temperature of 56°C.

### Protein analysis

The supernatant required for the alpha-amylase reporter enzyme assay was obtained by centrifugation of 2 ml culture at 10000 x g for 5 min at 4°C. The resulting supernatant was transferred to a new tube and stored at -20°C until further processing. The amylase activity was determined by use of the Ceralpha Assay (Megazymes, Wicklow, Ireland). To stay within the linear range, 1:200 dilutions in the suggested buffer were prepared. Measurements were done with a Tecan infinite M200 plate reader.

For the cytosolic proteome analysis, 8 ml of cells were harvested by centrifugation and washed twice with TE-buffer (10 mM Tris-Cl, pH 7.5, 1 mM EDTA) and finally resuspended in 1 ml TE with 2 mM PMSF (Carl Roth, Karlsruhe, Germany). Samples were transferred to cryotubes (Sarstedt, Nümbrecht, Germany) filled with 0.25 ml glass beads (Sartorius AG, Göttingen, Germany) and the cells were disrupted by bead-beating for 30 seconds at 6.5 m/s. After cooling for 5 min on ice, this procedure was repeated twice. Cell debris was removed by 30 min centrifugation at 17000 x g. The supernatant was stored at -20°C. 5 μg of protein mixture were separated by SDS-PAGE [35] and stained with silver-blue Coomassie [36].

The bands were excised using a sterile scalpel and transferred into 96 well micro titer plates (Greiner bio one, Frickenhausen, Germany). The tryptic digest with subsequent spotting on a MALDI-target was carried out automatically with the Ettan Spot Handling Workstation (Amersham Biosciences, Uppsala, Sweden). The gel pieces were washed twice with 100 μl of a solution of 50% CH_3_OH and 0% 50 mM NH_4_HCO_3_ for 30 min and once with 100 μl 75% CH_3_CN for 10 min. After drying at 37°C for 17 min 10 μl trypsin solution containing 4 μg/ml trypsin (Promega, Madison, WI, USA) was added and incubated at 37°C for 120 min. For extraction gel pieces were covered with 60 μl 0.1% TFA in 50% CH_3_CN and incubated for 30 min at 40°C. The peptide-containing supernatant was transferred into a new microtiter plate and the extraction was repeated with 40 μl of the same solution. The supernatants were completely dried at 40°C for 220 min. The dry residue was resuspended in 0.9 μl α-cyano-4-hydroxy-cinnamic acid matrix (3.3 mg/ml in 50%/49.5%/0.5% (v/v/v) CH_3_CN/H_2_O/TFA) and 0.7 μl of this solution was deposited on the MALDI target plate. The samples were allowed to dry on the target 10 to 15 min before measurement by MALDI-TOF mass spectrometry.

The MALDI-TOF measurement was carried out on the AB SCIEX TOF/TOF™ 5800 Analyzer (ABSciex /MDS Analytical Technologies, Darmstadt, Germany). This instrument is designed for high throughput measurement, being automatically able to measure the samples, calibrate the spectra and analyze the data using the TOF/TOF™ Series Explorer™ SoftwareV4.1.0. The spectra were recorded in a mass range from 900 to 3700 Da with a focus mass of 1700 Da. For one main spectrum 25 sub-spectra with 100 shots per sub-spectrum were accumulated using a random search pattern. If the autolytical fragment of trypsin with the mono-isotopic (M + H) + m/z at 2211.104 reached a signal to noise ratio (S/N) of at least 40, an internal calibration was automatically performed as one-point-calibration using this peak. The standard mass deviation was less than 0.15 Da. If the automatic mode failed (in less than 1%), the calibration was carried out manually. The five most intense peaks from the TOF-spectra were selected for MS/MS analysis. For one main spectrum 20 sub-spectra with 125 shots per sub-spectrum were accumulated using a random search pattern. The internal calibration was automatically performed as one-point-calibration with the mono-isotopic arginine (M + H) + m/z at 175,119 or Lysine (M + H) + m/z at 147,107 reached a signal to noise ratio (S/N) of at least 5. The peak lists were created by using GPS Explorer™ Software Version 3.6 (build 332) with the following settings for TOF-MS: mass range, 900–3700 Da; peak density, 20 peaks per 200 Da; minimum S/N ratio of 15 and maximal 65 peaks per spot. The TOF-TOF-MS settings were a mass range from 60 to Precursor - 20 Da; a peak density of 50 peaks per 200 Da and maximal 65 peaks per precursor. The peak list was created for an S/N ratio of 10. All peak lists were analysed using Mascot search engine version 2.4.0 (Matrix Science Ltd, London, UK) with a specific user sequence database and specific *B. subtilis* and *B. licheniformis* databases.

### ^1^H-NMR for extracellular metabolite analysis

2 ml cell culture was rapidly sterile filtered into a 2 ml tube and stored at -20°C. 400 μl of the extracellular sample was buffered to pH 7.0 by addition of 200 μl of a sodium hydrogen phosphate buffer (0.2 mM [pH 7.0], including 1 mM TSP)) made up with 50% D_2_O to provide a nuclear magnetic resonance (NMR)-lock signal. The samples were measured using 1H-NMR (600.27 MHz) at a nominal temperature of 310 K (Bruker AVANCE-II 600 NMR spectrometer, Bruker Biospin GmbH, Rheinstetten, Germany) as described previously [37]. AMIX 3.9 was used for data processing and analysis.

### Software

Graphs were created using R [38] and, where applicable, with the ggplot2 package [39]. Graphs for metabolites were created with VANTED V2.1.3 [40]. Genome analysis was done with Geneious version 5.6.2 created by Biomatters (Auckland, NZ) available from http://www.geneious.com. Free Gibbs-Energy (-∆G) of mRNA structures was determined with UNAfold [41].

## Competing interests

The authors declare that they have no competing interests.

## Authors’ contributions

JK has devised and carried out most of the experiments and written the manuscript. IP has transformed the *B. subtilis* ACE mutant and has carried out preliminary cultivations and RNA analyses. HM and ML have carried out the metabolomic investigation. DA has performed the MS-analysis and provided the MS-methodology description for the manuscript. AE has provided information about primer extension experiments of the *aceBA* operon. TS conducted the research, analyzed data and revised the paper. All authors read and approved the final manuscript.
